# Ectoparasite Fauna of Rodents and Shrews with Their Spatial, Temporal, and Dispersal along a Degradation Gradient in Mabira Central Forest Reserve

**DOI:** 10.1155/2023/7074041

**Published:** 2023-10-28

**Authors:** Waswa Sadic Babyesiza, Joseph Mpagi, James Ssuuna, Sisiria Akoth, Abdul Katakweba

**Affiliations:** ^1^Africa Centre of Excellence for Innovative Rodent Pest Management and Biosensor Technology Development (ACE IRPM&BTD), Uganda; ^2^Department of Wildlife Management, Sokoine University of Agriculture, Morogoro, Tanzania; ^3^Department of Zoology, Entomology and Fisheries Science, Makerere University, Uganda; ^4^Department of Microbiology and Immunology, Busitema University, Uganda; ^5^Institute of Pest Management, Sokoine University of Agriculture, Morogoro, Tanzania

## Abstract

Ectoparasites like fleas, mites, and ticks that are key carriers of harmful pathogens such as viruses, bacteria, cestodes, and nematodes live on rodents and shrews. It should be noted that rodents' ecological adaptability makes them suitable as parasite hosts. The main objective of the study was to determine the ectoparasite assemblages in rodents and shrews along a degradation gradient, while comparing infestation levels in different habitats with varying levels of degradation. The study was conducted in Mabira Central Forest Reserve. Ectoparasites were collected following rodent and shrew removal trapping which was done using Sherman's traps set along transects of 200 meters in three habitat strata that included adjacent forest habitats, degraded forest edge, and regenerating forest interior. Data was collected intermittently with a break every two months for one year from November 2018 to December 2019. A total of 1411 rodents and shrews were collected, yielding a total of 5692 ectoparasites from 22 host species (17 rodents and 5 shrews). The most prevalent group of ectoparasites was mites followed by fleas, lice, ticks, and earwig. Ectoparasite prevalence significantly differed depending on hosts species (*P* = 0.001) and host age (*P* = 0.022), but not host sex (*P* = 0.78), while mean infestation significantly varied basing on host species (*P* = 0.001), host sex (*P* = 0.001), season (*P* = 0.001), and habitat (*P* = 0.001). Prevalence (*P* = 0.001) and mean infestation (*P* = 0.001) significantly varied across studied habitats. The study has emphasized the significance of *Praomys jacksoni* and *Hylomyscus stella* as significant hosts for mites and *S. congicus* as a significant host for fleas. Additionally, environment and host characteristics have a bearing on prevalence and infestation of ectoparasites with habitat degradation playing a significant role in the occurrence of ectoparasites, thereby emphasizing its contribution to zoonotic outbreaks.

## 1. Introduction

Uganda has a rich and diverse fauna of rodents and shrews [1–3]. These shrews and rodents serve as hosts for various ectoparasites within the phylum Arthropoda [4] that are possible zoonotic disease vectors [5, 6]. The ectoparasites of the rodent and shrew fauna of the African tropics are also little understood [4]. Fleas and ticks are some of the most well-known rodent and shrew ectoparasites [4, 7]. These parasitic ectoparasites serve as secondary hosts or reservoirs for a number of infections, including *Bartonell*a spp., *Rickettsia* spp., and *Yersinia pestis* [8]. Consequently, ectoparasites vector pathogens to their hosts as they feed or defecate [9].

Rodents are hosts for a variety of ectoparasites mostly due to their vast range of lifestyles, adaptable behavior, and capacity to adjust to environmental changes [10]. It should be noted that various rodent and shrew species occupy varying habitats which influences the diversity and abundance of ectoparasites they host [11, 12]. High global diversity of rodent and shrew species and ectoparasites that infest them results in a wide range of associations; these associations are affected by copious characteristics of the host (sex and age), parasite species, and other biotic and abiotic factors [10].

Buchholz and Dick [13], noted that establishment of a successful host parasite association is associated with the likelihood of an ectoparasite encountering a host. This phenomenon was however conceptualized by Combes [14, 15] and Kołodziej-Sobocińska [16], as an encounter filter. The encounter filter eliminates possible hosts that a parasite would not encounter because of the host's or the parasite's ecological or behavioral traits. This motivates the parasite's ecology and evolution to target the hosts that it is most likely to encounter [16]. Furthermore, Bitam et al. [17] highlighted that ectoparasites tend to occupy microhabitats of the host species where they wait until a suitable host is present, consequently allowing the parasite to colonize the host. Social behavior of the host species can also facilitate transfer of ectoparasites from one host to another [18].

Although not given much attention, through their direct and indirect effects on their hosts, the various species of ectoparasites have been and continue to be a threat to humans and their livestock [19, 20]. Over the years, the associations between ectoparasites and their hosts have been subjected to a number of dramatic changes with many hosts becoming synanthropic. Most of the changes are mediated by human activities, which have resulted in a substantial shift in the nature of many parasite-host relationships [9, 19, 21].

With the rapid increment in human population worldwide, many natural and seminatural habitats inside and outside protected areas have been encroached on for settlement and other developments. Coupled with this, increased rates of human movements worldwide and increasing urbanization have the potential to affect the distribution of ectoparasites with pathogenic microorganisms and their hosts. Paramasvaran et al. [21] indicated that most ectoparasites are believed to have lived in close association with their animal hosts in stable habitats which have for a long time been in equilibrium with the climate and soil, undisturbed by humans. Once this equilibrium is altered due to anthropogenic activities, there is a great danger of zoonotic infections being transmitted to humans and their livestock [19].

Mabira Central Forest Reserve (MCFR) is the largest forest reserve in Central Uganda [22] and managed by Uganda National Forestry Authority (NFA) as a Central Forest Reserve (CFR). According to Howard [23] and Mulugo et al. [24], MCFR is considered “secondary regenerating,” in which the most dominant vegetation represents subculmination communities, heavily influenced by man through continued excess illegal resources use from the forest and encroachment. The status of forests in Uganda is a result of continued deforestation [24, 25] with a deforestation rate of 1.9% the highest in East Africa [25]. Most of the forest land cleared especially on private land has been converted into settlements, gardens, and plantations [24]; this has greatly increased the interaction between humans and their livestock with wildlife especially rodents and shrews Mulugo et al. [24].

Mabira Central Forest Reserve (MCRF) is located between metropolitans of Lugazi, Mukono, Jinja, and Kampala, which put the forest under immense pressure for resources leading to changes in forest habitat structure. This coupled with the unpredictable weather patterns has a significant impact on the biological dynamics of fauna hosts and their associated ectoparasites. It is, however, unclear how these factors will interact over time to affect the dynamics of ectoparasite communities. The study is aimed at determining the ectoparasite assemblages in rodents and shrews along a degradation gradient, while assessing how habitat characteristics, host characters, and seasonality affect ectoparasite prevalence and infestation in rodents and shrews.

## 2. Methods

### 2.1. Study Area

The study was conducted in MCFR in Uganda (0° 26′ 14.28^″^ N, 32° 57′ 14.31^″^ E, 1179 m). Locally, the word “Mabira” translates as great forest. Mabira Central Forest Reserve is located in Central Uganda, 57 km from Kampala, the capital city of Uganda, and 26 km from Jinja, formerly the industrial town of Uganda. The reserve covers 306 km^2^ and is one of the important protected areas (PAs) in Uganda with 47% of Uganda's timber species [24, 25], including three tree species (*Milicia excelsa* (NT), *Entandrophragma Angolense* (VU), and *Warburgia ugandensis* (VU)) listed by the International Union for Conservation of Nature (IUCN) [25, 26].

### 2.2. Study Design

**Figure 1:**
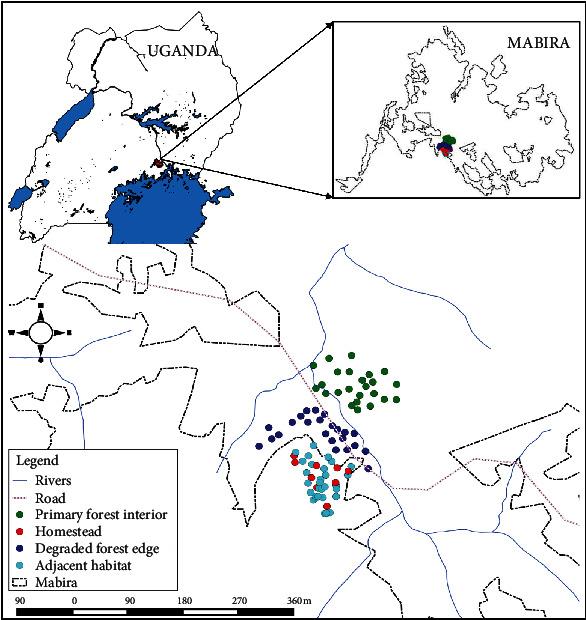
Map of a stratified sampling area representing a degradation gradient in Mabira Central Forest Reserve, Central Uganda.

From November 2018 to December 2019, the study was conducted in the village of Namusa in the MCFR. Data were intermittently collected (with a one-month break after two months of data collection, totaling 10 sampling regimes) along a gradient of habitat degradation that included a primary forest interior, a degraded forest edge, adjacent habitats, and homesteads (Figure 1 and Table 1). For every sampling regime, three randomly selected transect replicates were set in each of the subjectively selected sites (Figure 1 and Table 1).

### 2.3. Trapping and Identification of Rodents and Shrews

Rodents and shrews were captured using transect lines established in each arbitrary selected sampling site. For every sampling regime, three transects of 200 m were set in each of the sampling sites. Each transect had 20 sampling stations spaced 10 m apart; at each sampling station, a Sherman removal trap was set baited with a combination of peanut butter, maize flour, ripe bananas, and silver fish [28]. Each transect line was left in the same spot for three nights, checked every morning and evening to remove any captured specimen. Specimens were then processed by recording their age, sex, and morphometric measurements (hind foot, head and body, tail, ear length (mm), and weight (g)) and given a unique field number. Utilizing morphometric data, specimens were identified down to species level [1, 3, 29, 30]. Rodent and shrew identifications were confirmed by sequencing of partial mitochondrial cytochrome b gene (i.e., DNA barcoding) from 96% ethanol-preserved samples at the Institute of Vertebrate Biology (IVB) of the Czech Academy of Sciences. Obtained sequences were compared with the sequences in GenBank and further unpublished sequences in the database of IVB. The barcoding protocol (i.e., used primers, PCR conditions, and sequencing) is described in Bryja et al. [31].

### 2.4. Examination of Ectoparasites

Specimens were anesthetized using halothane, and the fur of each anesthetized rat or shrew was combed using a tooth brush to remove any ectoparasites and place them on an enamel tray. When ticks and mites were difficult to remove by combing, fine forceps were employed to extract them from the skin of the rodents and shrews.

Each cloth bag in which the rat was housed was turned upside down over the enamel tray to collect ectoparasites that had become loose and dropped in the bag. With the aid of a hand lens, the contents of the enamel tray were carefully examined. Any ectoparasites discovered were then retrieved using a small, moistened paintbrush and placed in individual cryovials that contained 70 percent alcohol [32, 33].

All fleas, lice, mites, and ticks were collected, counted, and stored in 70% ethanol. For taxonomic identification, ectoparasites were removed from the alcohol and rinsed in water. They were next placed in lactophenol (a clearing agent) for up to 5 days at room temperature. Cleared specimens were washed once in distilled water and mounted in Hoyer's medium [34].

After mounting, the parasites were examined under a light microscope at magnifications of ×10 for large parasites (ticks, fleas, and mites) and ×40 for small parasites (lice). Published identification instructions [35–40] served as the basis for identification. Mite expert Barry OConnor from the University of Michigan Museum of Zoology was consulted to help in the identification of cryptic mite species.

### 2.5. Data Analysis

Ectoparasite mean abundance in rodents and shrew was calculated from the formula = number of ectoparasites collected from examined rodents and shrews/total number of examined rodents and shrews. The constituent ratio (*C*) and prevalence (*P*) were calculated according to Xing-Yuan et al. [41], using the following formulas: *C* = *N*_*i*_/*N* × 100%, where *N*_*i*_ represents the number of individual ectoparasites in group *i* (total ectoparasites, ticks, fleas, lice, and mites) and *N* represents the total number of ectoparasites, and *P* = *H*_*i*_/*H* × 100%, where *H* represents the total number of rodents and shrews sampled and *H*_*i*_ represents the number of individual rodent and shrew hosts parasitized by group *i*. Mean infestation = total number of ectoparasites on a specific rodent or shrew species/total number of rodents or shrews of the same host [42].

In order to have an insight of the association between hosts and their parasites, we used Kendall's correlation coefficient. This coefficient is a nonparametric measure of the strength and direction of association that exists between the two variables [43, 44].

All the data collected was also entered in Excel processed and exported to Stata (Stata 14) for analysis. Since our original continuous data (mean infestation and parasite prevalence) did not follow the bell curve, we log transformed it to make it as “normal” as possible in order to validate the statistical analysis results. We used one-way ANOVA to test whether the parasite prevalence and mean infestation vary depending on host sex, host age, habitat, and seasonality. To further examine the strength of explanatory variables in the mean infestation or parasite prevalence, we perform a natural log-level regression with dummies (multiple linear regression with dummies). The regression model was specified as

(1) $$\begin{array}{c} \text{Ln}\left(Y_{i}\right)=\beta_{0}+\beta_{i}^{\prime }X_{i}+\varepsilon_{i}, \end{array}$$

where Ln(*Y*_*i*_) is the log-transformed mean infestation/parasite prevalence, **β**_**i**_′ are the vector regression coefficients, **X**_**i**_′ are the vector of covariates corresponding to each of the rodents, and *ε*_*i*_ are the errors which represent the unexplained variations in the log-transformed mean infestation/parasite prevalence. We therefore log transformed the dependent/response variable simply because its distribution did not follow a normal distribution. Exponentiate the coefficient, subtract one from this number, and multiply by 100, and this gives the percent increase (or decrease) in the response for every one-unit increase in the explanatory/dummy variables (Table 2).

### 2.6. Ethical Clearance

This study was first approved by Sokoine University of Agriculture Directorate of Post Graduate Studies (Ref. no: PFC/D/2017/0004) (20 Feb 2018), which was followed by other ethical approvals in Uganda from relevant authorities: Uganda Wildlife Authority (UWA) (Ref. no: UWA/COD/96/02) (26 April 2018); National Forest Authority (NFA) (Ref. no: NFA/N/2.1/17, License no: 291) (17 August 2018); and Uganda National Council for Science and Technology (UNCST) (Ref. no: NS54ES) (30 October 2018).

## 3. Results

### 3.1. Occurrence of Ectoparasites in Rodent and Shrew Hosts of Mabira Central Forest Reserve

**Figure 2:**
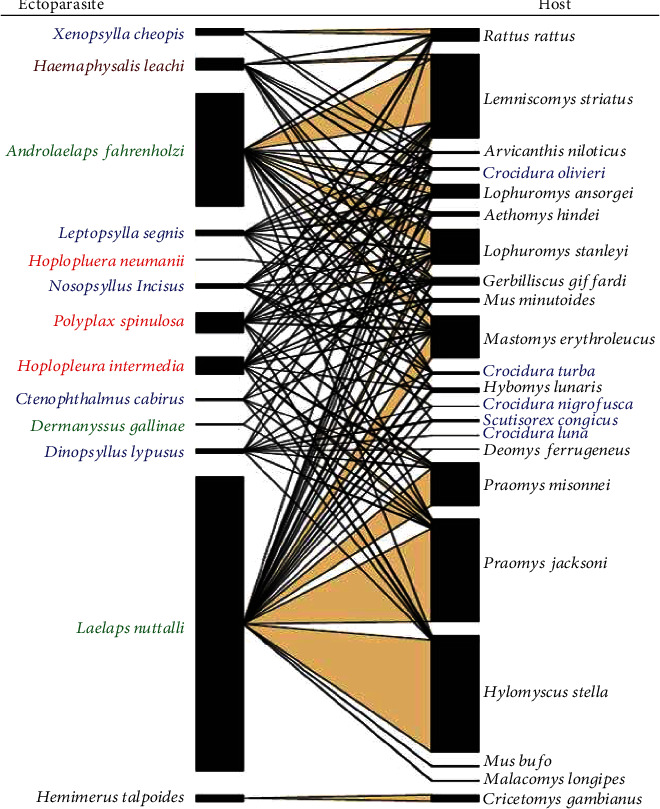
Plot web of ectoparasites and their hosts in Mabira central forest. Key, ectoparasite side: blue = fleas, green = mites, red = lice, and black = earwig. Host side: blue = shrews and black = rodents.

The trapped animals yielded a total of 5692 ectoparasites from 22 species, that is, 17 rodents and 5 shrews (Table 3). There were three species of mites, five species of fleas, three species of lice, one species of tick, and one earwig among them, altogether 13 ectoparasites (Table 3). Out of the 1411 rodents and shrews identified in the three sampling areas, only 883 exhibited infestations by ectoparasites. Among the captured rodents, only three species (Oenomys hypoxanthus, Grammomys macmillani, and Mus triton) were found without any ectoparasite infestations. Conversely, all other documented species of rodents and shrews had ectoparasite infestations. Notably, only four species were infested by a solitary ectoparasite species (see Figure 2).

All examined rodents and shrews were found to have more than one species of ectoparasite with rodents *Praomys jacksoni*, *Hylomyscus stella*, and *Lemniscomys striatus* being important hosts for a wide range of ectoparasites (Table 3 and Figure 2). *Mus minutoides*, *Crocidura luna*, *Malacomys longipes*, and *Cricetomys gambianus* were infested by only one parasite type (Table 3). Only two species of ectoparasite, the earwig *Hemimerus talpoides* (*Cricetomys gambianus*) and lice *Hoplopluera neumanii* (*Gerbilliscus validus*), were found to be with only one rodent host (Figure 2).

There is an observed level of association between rodents and shrews and the type of parasite, though not very strong due to the small value of Kendall's tau − *b* = −0.0326. The importance of each host species for a given ectoparasite is however illustrated in Figure 2. For example, you can observe that most *Laelaps nuttalli* mites were recovered from *H. stella* and *P. jacksoni*. *Lophuromys stanleyi* and *Lemniscomys striatus* are important hosts for the mite *Androlaelaps fahrenholzi*, the latter being also important for the tick *Haemaphysalis leachi*. *Rattus rattus* is a major host for the flea *Xenopsylla cheopis* having 96% of all recovered specimens of this flea (Table 3 and Figure 2), while *Cricetomys gambianus* is a major host for the earwig *Hemimerus talpoides* (Table 3 and Figure 2).

The mite *Laelaps nuttalli* occurred in larger numbers representing 60.8% of all the recovered ectoparasites (Table 3). Of all the fleas recovered, *Xenopsylla cheopis* was the most abundant (1.25%); it was however recovered from only three hosts. Fleas *Dinopsyllus lypusus* (0.88%) and *Nosopsyllus Incisus* (0.9%) were recovered from most hosts (Table 3) showing no particular host specificity even though the former was hosted most in the forest environments.

Different habitats yielded varying percentages of ectoparasite species (Table 4). The mite *Androlaelaps setosus* (54%) was most prevalent in adjacent habitats, while *Laelaps nuttalli* was the most abundant in degraded forest edge (83%) and primary forest interior (88.1%). Among lice, *Hoplopluera neumanii* was recovered from one habitat (adjacent habitat), but *Polyplax spinulosa* and *Hoplopleura intermedia* were found in all habitats, with the former being more abundant (14.7%) in homesteads (Table 4). Flea abundance was highest in adjacent habitats, followed by degraded forest edge with *Leptopsylla segnis* showing the highest abundance in the two habitats and least in primary forest interior with *Dinopsyllus lypusus* (1.6%) showing the highest abundance (Table 4).

The most prevalent group of ectoparasites in rodents was mites (61%, *n* = 716) followed by fleas (11%, *n* = 146) and lice (7.4%, *n* = 98), while in shrews, fleas were the most prevalent (21.4%, *n* = 18) compared to all other parasites (Table 5). Mites represented the highest ectoparasite index in rodents while in shrews, fleas had the highest ectoparasite index, but mean abundance for mites was equally high in rodents and shrews compared to other parasite groups (Table 5).

The prevalence of several parasite types within rodent and shrew species was low. High prevalence of mites was found in rodents *Hylomyscus stella* (17.61%) and *Praomys jacksoni* (16.16%), as well as in shrews *Crocidura turba* (4.76%) and *Crocidura olivieri* (4.76%) (Table 6). Overall prevalence of fleas was highest among shrews *Scutisorex congicus* (7.14%) and *Crocidura olivieri* (5.95%) with only *Rattus rattus* among rodents recording a prevalence above 2% (Table 6). *Crocidura olivieri* also showed high prevalence (8.33%) for ticks, much higher than any shrew or rodent species recorded. Other notable observations are rodent species *Mastomys erythroleucus* and *Lophuromys stanleyi* from which all ectoparasite species besides *Xenopsylla cheopis* (Flea) and *Hemimerus talpoides* (Earwig) were recovered (Table 3).

### 3.2. Effect of Host and Environmental Characteristics on Ectoparasite Prevalence and Mean Infestation

Ectoparasite prevalence significantly differed depending on host species (*P* = 0.001) and host age (*P* = 0.022), but not host sex (*P* = 0.78). Furthermore, prevalence varied greatly depending on the kind of habitat (*P* = 0.001) but not according to the season (*P* = 0.075) (refer to Table 7).

As a result, we only took into account host age, habitat, and season, when looking at the potential determinants of prevalence. It was noted that being a subadult increases prevalence by 8.6% compared to juveniles and reduces by 4.8% among adults (Table 8). Prevalence of ectoparasites on rodents and shrews habiting homesteads reduced by 6.2%, while in degraded forest edge and primary forest interior, it significantly reduced by 54.3% and 56.5%, respectively, compared to those habiting adjacent habitats. Also during the wet season parasite, prevalence significantly reduced by 18.5% compared to the dry season (Table 8).

Ectoparasite mean infestation significantly varied basing on host species (*P* = 0.001), host sex (*P* = 0.001), season (*P* = 0.001), and habitat (*P* = 0.001) but not host age (*P* = 0.788) (refer to Table 7).

Therefore, to examine the possible predictors of mean infestation, we considered only host sex, habitat, parasite type, and season. And it was ascertained that being a male increases the mean infestation by 16.2% as compared to females. Compared to adjacent habitats, mean infestation in degraded forest edge significantly reduced by 13.7% while in primary forest interior, it reduced by 6.4%, and in homesteads, it increased by 2.4% (Table 9). In the wet season, mean infestation of ectoparasites on rodents and shrews significantly reduced by 12% as compared to the dry season (Table 9).

## 4. Discussion

Muridae are an important group of rodent hosts that have diversely adapted to habiting various habitats even following anthropogenic changes [45]. *Praomys jacksoni* and *Hylomyscus stella* were the most important hosts in the forested environment while *L. striatus* was an important host in adjacent habitats; this is consistent with findings of Mawanda et al. [46], who found *Praomys jacksoni* to have the highest infestation of ectoparasites in Bwindi forest reserve. This could be explained by the high abundance of these species in their preferred habitat which increases the chance of them encountering ectoparasites [47].

The study revealed that mites were the most abundant ectoparasites followed by fleas, lice, and lastly the ticks. Most species of mites show a narrow host specificity being able to infest a wide number of hosts which tends to perpetuate their numbers in any given habitat [46]. Their ability to stay long between blood meals so long as they have mite islands (James H. [48]) helps them to survive until they find a suitable host. These observations concur with those of Mawanda et al. [46] in Uganda, Shayan et al. [49] in Iran, and Paramasvaran et al. [21] in Malaysia who found mites to be the predominant ectoparasites in their studies.

The mites of the genus *Laelaps* (Laelapidae) are common ectoparasites of small mammals, particularly rodents. From this study. *Laelaps nuttalli*, a hematophagous parasite of humans and rodents [50], was found to be the most abundant ectoparasite species hosted by majority of rodents and shrews (Table 3 and Figure 2). This implies low host specificity by *L. nuttalli* and ability to utilize a variety of mite islands including humans.

*Androlaelaps fahrenholzi* was the most dominant mite in habitats outside forest compared to *Laelaps nuttalli* which was dominant in forested environments. However, these species have been documented to occupy a wide range of habitats [46, 49, 51]. It can therefore be deduced that these are widespread species with a similar host range. In such a scenario, habitat partitioning tends to occur as explained by Wisheu [52]. Here, the dominant, intolerant species (*Laelaps nuttalli*) occupies the preferred segment of the habitat gradient (forest with high densities of hosts) while the tolerant subordinate species (*Androlaelaps fahrenholzi*) occupies habitat with suboptimal levels of resources in this case adjacent habitats.

Species from the genus *Laelaps* have been implicated in being reservoirs of *Rickettsia* spp. [50]. The high abundance of *L. nuttalli* perpetuated in forested areas, coupled with its ability to occur in peridomestic rodents and potential to vector *Rickettsia*, highlights its importance in the public health of people around MCFR. With continued habitat degradation, this risk becomes more apparent as peridomestic rodents become more abundant and dominant *L. nuttalli* can easily switch its preferred hosts.

The recording of *Dermanyssus gallinae*, a known galliform mite on two rodent species (*P. Jacksoni* and *L. stanleyi*) occupying different habitats, highlights possible interactions between commensal rodents that interact with hens and wild rodents and ultimately humans in the same environment. This is exacerbated with the ongoing forest encroachment and consequential degradation increasing interactions between peridomestic and wild rodent and shrew species. These interactions could be a significant channel for the transmission of infections carried by *D. gallinae*, thus posing a health risk to communities surrounding MCFR.

Polyparasitism which refers to the presence of many species on a single host [47] was exhibited by most species particularly in *P. jacksoni* and *H. stella* habiting forested ecosystems, *L. stanleyi*, *Lemniscomys striatus*, and *Mastomys erythroleucus* in adjacent habitats. This implies that the ecology of these rodents provides favorable environment for the survival and proliferation of these parasites. According to Obiegala et al. [47], polyparasitism leads to higher abundance as well as higher prevalence rates for most ectoparasite species in rodents and shrews.

The plethora abundance of *X. cheopis* on *Rattus rattus* observed in this study concurs with the findings of Moore et al. [53] and Eisen et al. [54], in Uganda, whose results all showed great association between *Rattus rattus* and *X. cheopis*. The recording of *X. cheopis* which is the most efficient vector of *Y. pestis* [55] around homes raises the risk of plague outbreak around MCFR. Other fleas recorded were *Ctenophthalmus cabirus*, *Dinopsyllus lypusus*, *Nosopsyllus incises*, and *Leptopsylla segnis*, which are also important zoonotic vectors of bacteria such as *Bartonella* spp. and *Rickettsia* spp. Even though not the primary hosts, *Dinopsyllus lypusus* and *Leptopsylla segnis* have been documented to vector *Yersinia pestis* by Bai et al. [56] and Jones et al. [57], in Uganda. These flea species were collected from domestic, peridomestic, and wild rodents suggesting increased interactions between peridomestic and wild rodent and shrew species as a result of forest habitat degradation. This creates a risk of transfer of zoonotic pathogens from the wild into communities and vice versa as in Jones et al. [57].

Widely active plague foci exist in Southern and Eastern Africa, Uganda, inclusive. In these areas, *Xenopsylla* and *Dinopsyllus* species are the principal flea vectors of wild rodent hosts while *X. cheopis* and *X. brasiliensis* are the principal flea species involved in transmission among commensal rats and humans [58]. *Rattus rattus* is one of the primary natural hosts of *Xenopsylla cheopis* [21, 59, 60] which explains why 95% of all recorded *X. cheopis* from this study were recovered from *Rattus rattus*. *Xenopsylla cheopis* is a parasite of many mammalian species, including *Rattus rattus* and humans. Because of its parasitic nature, *Xenopsylla cheopis* is a vector for pathogens such as plague bacilli, *Yersinia pestis*, and murine typhus, *Rickettsia typhi*. Mabira Central Forest Reserve has a history of zoonotic outbreaks where in the past, around 1914 people who had settled in the enclaves of the forest were driven out by outbreaks of plagues [61].

The process of host selection by ectoparasite follows rigorous adaptations to the biotic (morphological and biological) and abiotic (host surroundings) characteristics of the host [18]. This process is so complex occasionally resulting in some parasites choosing to infest one host. Notably is the parasite *Hemimerus talpoides* which was only recovered from *Cricetomys gambianus*, as also reported by Ashford [62]. This kind of host specificity can only be attributed to generations of adaptations and coevolution between *Cricetomys gambianus* and *Hemimerus talpoides* [63].

With regard to rodents and shrews, species richness and diversity have been known to follow the intermediate disturbance hypothesis which states that species richness is at maximum with intermediate levels of disturbance [64]. Similar observations were observed in our data set with species richness and diversity being highest along the degraded forest edge compared primary forest interior (Table 4). When it came to rodent and shrew ectoparasites, however, the highest mean infestation was observed in the primary forest interior. This could be explained with the dilution effect, which implies that where species vary in susceptibility to infestation by parasites, higher diversity leads to lower infestation prevalence in hosts [65, 66]. This observation may however be explained by other factors such as habitat characteristics, seasonality, or a combination of all [51].

The difference in infestation levels of rodents and shrews can be explained by the ecology and behavior of the two small mammal groups. Shrews are mainly insectivorous [67] while most rodents can be grouped as herbivores [68]. With their diet consisting more of arthropods, it can be argued that shrews can potentially prey on their ectoparasites unlike rodents, hence low infestation. Some rodent species also occur in high densities (*P. jacksoni*) Mizerovská et al. [69], which increases their chances of encountering ectoparasites [16].

The high prevalence of fleas on *S. congicus* can be explained by its ecology and behavior of using tunnels and burrows when feeding in its habitat as highlighted in Kasozi [69]. The study of Kreppel et al. [71] highlighted that fleas are known to prefer living in burrows and only crawl onto hosts in order to feed. The study also noted that animal burrow networks provide the necessary humidity for immature flea development and also lessen the harsh effects of weather above ground, creating conditions that are more suited to the growth and population dynamics of fleas. Therefore, it might be suggested that *S. congicus* picks up the many fleas as it moves through the tunnels and burrows.

*Crocidura olivieri*, another shrew with high prevalence of fleas, is a very adaptive shrew occurring in natural and modified habitats within its range [72]. This high adaptability implies that *C. olivieri* can cross various habitat strata interacting with various rodent while preying on some (personal observation), which increases its encounter rate with potential ectoparasites. It was also found to have a high prevalence of the only tick species recorded *Haemaphysalis leachi*. *H. leachi* primary hosts are dogs, but its larvae and nymphs usually infest common murid rodents [73]. The fact that *C. olivieri* can also be commensal leaving in fallows and homesteads increases chances of picking up larvae and nymphs of *H. leachi*. This makes *C. olivieri* an important player in the transmission of the protozoan *Babesia canis* to dogs, causing canine babesiosis, and the bacterium *Rickettsia conorii* which causes tick typhus in humans [73].

### 4.1. Predictors of Ectoparasite Prevalence and Infestation in Rodents and Shrews

From our results, it has been construed that both host and environmental characters affect ectoparasite prevalence and infestation which is consistent with studies of Hammond et al. [74], in USA, and Shilereyo et al. [51], in Serengeti, Tanzania. Among host characters, age is much more an efficient predictor of ectoparasite prevalence compared to sex as in Marcela [75]. Compared to juveniles that stay close to their nesting sites, adults range far and wide in search of food and mates and as such have high chances of parasite encounter compared to juveniles. Even though host sex was found to be a poor predictor of parasite prevalence, previous studies have found males to have a higher prevalence compared to females. Matthew and Carl [42] hypothesized that males would have higher prevalence and mean intensity of flea and tick infestation due to male rodents having generally larger dispersal areas. Bitam et al. [17], from their study on fleas across the world, noted that most ectoparasites rely on the host coming to the parasite instead of the parasite searching out a host; active hosts that cover larger spatial areas would likely encounter parasites more frequently. This would be true in places with resource scarcity, but in places like forests with high host densities and high resources, availability dispersal in both sexes might be almost the same which explains the observations in our study.

Environmental factors are also very important in predicting parasite prevalence and infestation as shown from our results. Seasonality affects ectoparasite prevalence and intensity as observed by Kordiyeh et al. [76]. From this study, it was observed that ectoparasite infestation and prevalence were high during the dry season which is consistent with findings of Kordiyeh et al. [76], in Iran, and Welegerima et al. [77], in Ethiopia. This can be explained by the assumption that parasite prevalence and infestation follow host abundance. In this case, even though most rodents breed throughout the year, this can only be true in scenarios where resources are abundant. However, peaks of breeding are usually during the rainy season [78, 79]; this then implies that all newborns reach adulthood during the dry season, hence bursts in rodent and shrew abundance and consequently their ectoparasites because of the high encounter rates. Habitat variability also affected parasite prevalence and infestation, with the highest prevalence recorded in the adjacent habitats compared to forest edge and primary forest interior. Adjacent habitats represented areas comprised of fallows, gardens, and plantations; these are known to harbor low diversity compared to forested habitats but high densities of rodents and shrews and associated ectoparasites [51, 79, 80]. The high species diversity in forested ecosystems also creates a dilution effect, hence reducing infestation and prevalence. Only homesteads had a high ectoparasite infestation compared to adjacent habitats, which could be attributed to communal congregations of commensal rodents and occurrence in high abundances [81].

## 5. Conclusion

Rodents and shrews captured in and around MCFR are a host to a wide range of ectoparasites which vector various pathogens, some of which are potential causative agents of zoonoses. The study has highlighted the importance of *P. jacksoni and H. stella* as important hosts for assorted ectoparasites especially *Laelaps nuttalli*, one of the known vectors of *Rickettsia* spp. We can also conclude that most abundant rodent species in different habitats exhibit polyparasitism perpetuating the occurrence of most ectoparasites. As observed in other studies, *Rattus rattus* is the major host of the plague flea *X. cheopis* with other peridomestic rodents and shrews like *Arvicanthis niloticus* and *Crocidura olivieri* coming into play. This emphasizes the need of managing populations of commensal and peridomestic rodents to reduce risks of epizootic outbreaks. With continued habitat degradation, peridomestic rodents and shrews will become more abundant, leading to host switching by the less host-specific mites from forest rodent hosts to peridomestic rodents creating a public health risk. We can also conclude that *S. congicus* is an important reservoir of fleas especially *Dinopsyllus lypusus*.

Even though host age, seasonality, and nature of habitat have been highlighted as good predictors of ectoparasite prevalence and infestation, intraspecies ecology, behavior, and microhabitat characteristics need to be investigated for better and accurate insights in rodent ectoparasite prevalence and associated zoonotic risks. In addition to the above recommendation, the biology and behavior of different parasite types in different habitats need to be investigated as different microhabitat characteristics might stimulate evolution of certain behavioral strategies even in similar parasites but occupying different habitats.

## Figures and Tables

**Table 1:** Summary of habitat description and study design.

Habitat	Vegetation cover	Trapping effort for every trapping regime
Homesteads	These represented in and out human habitations; inside stores, kitchens, and gardens or bush nearby	Number of traps = 5 @ house Number of nights = 3

Adjacent habitat	Consisting of a mosaic of sugarcane plantations, fallows, and gardens in communities adjacent to the forest	Transect replicates = 3 Transect length = 200 m Number of traps = 40 Number of nights = 3

Degraded forest edge	Characterized by the prevalent presence of the invasive *Broussonetia papyrifera*, along with dense undergrowth and an open canopy, within the production zone primarily covering 33% (11,230 ha). *N* of the entire forest cover (30,000 ha)	Transect replicates = 3 Transect length = 200 m Number of traps = 40 Number of nights = 3

Primary forest interior	Dominated by *Funtumia africana* and other native large trees like *Celtis mildbraedii* and *Teclea nobilis*, characterized by a dense canopy and little undergrowth within the strict nature reserve zone, which encompasses 23% (7,350 ha). *N* of the entire forest cover (30,000 ha).	Transect replicates = 3 Transect length = 200 m Number of traps = 40 Number of nights = 3

*N* = Nabanoga et al. [27].

**Table 2:** Explanatory/dummy variable description.

Variables	Description	Coding
Habitat	Habitat of the host	0 = adjacent habitat 1 = degraded forest edge 2 = primary forest interior 3 = homestead

Sex	Sex of the host	0 = female, 1 = male

Season	Trapping season	0 = dry, 1 = wet

Age	Host age	0 = juvenile, 1 = adult

**Table 3:** Rodent and shrew species hosts and their associated ectoparasites in Mabira Central Forest Reserve.

		*Androlaelaps fahrenholzi (M)*	*Ctenophthalmus cabirus (F)*	*Dermanyssus gallinae (M)*	*Dinopsyllus lypusus (F)*	*Haemaphysalis leachi (T)*	*Hemimerus talpoides (E)*	*Hoplopleura intermedia (L)*	*Hoplopluera neumanii (L)*	*Laelaps nuttalli (M)*	*Leptopsylla segnis (F)*	*Nosopsyllus incisus (F)*	*Polyplax spinulosa (L)*
Rodents	*Aethomys hindei*	13 (1)			6 (12)					3 (0.1)	21 (34)		2 (0.8)
	*Arvicanthis niloticus*	15 (1)	2 (10)			1 (1)				3 (0.1)	1 (1.6)	1 (2)	6 (2.5)
	*Cricetomys gambianus*						75 (100)						
	*Deomys ferrugeneus*				1 (2)					0			
	*Gerbilliscus giffardi*	37 (3)				1 (1)		14 (7)	1 (100)	17 (0.5)	1 (1.6)	5 (10)	13 (5.3)
	*Hybomys lunaris*	4 (0.3)						5 (2.4)		32 (0.9)		8 (16)	
	*Hylomyscus stella*	4 (0.3)	1 (5)		2 (4)			22 (11)		1324 (38)			23 (10)
	*Lemniscomys striatus*	753 (57)			1 (2)	54 (42)		31 (15)		65 (2)	3 (5)	15 (29)	54 (22)
	*Lophuromys ansorgei*	92 (7.)				22 (17)		5 (2.4)		27 (1)	7 (11)	3 (6)	8 (3.3)
	*Lophuromys stanleyi*	220 (17)	2 (10)	1 (25)		12 (9)		8 (4)		90 (3)	15 (24)	8 (16)	46 (19)
	*Malacomys longipes*									8 (0.2)			
	*Mastomys natalensis*	131 (10)	5 (25)		1 (2)	13 (10)		15 (7		297 (9)	6 (10)	6 (11.8)	20 (8)
	*Mus bufo*									7 (0.2)			
	*Mus minutoides*	3 (0.2)				2 (2)		7 (3)		17 (0.5)			12 (5)
	*Praomys jacksoni*	7 (0.5)	9 (45)	3 (75)	15 (30)			59 (29)		1099 (32)	1 (1.6)		18 (7)
	*Praomys misonnei*		1 (5)		8 (16)			28 (14)		441 (13)			16 (6)
	*Rattus rattus*	29 (2)				12 (9)		3 (2)		3 (0.1)			25 (10)

Shrews	*Scutisorex congicus*	1 (0.1)			13 (26)					3 (0.1)			
	*Crocidura turba*	6 (0.5)			1 (2)	1 (1)				20 (0.6)	3 (5)	2 (4)	
	*Crocidura luna*				1 (2)								
	*Crocidura nigrofusca*				1 (2)					2 (0.1)		1 (2)	
	*Crocidura olivieri*	7 (0.5)				10 (8)		8 (4)		0	4 (6.4)	2 (4)	

	% abundance for each parasite	23.23	0.35	0.07	0.88	2.25	1.32	3.60	0.02	60.79	1.09	0.90	4.27

In parenthesis is the percentage abundance of each parasite on a host. Codes: M = mite; F = flea; T = tick; L = lice; E = earwig.

**Table 4:** Ectoparasites of rodents and shrews and the habitats they inhabit.

	Homestead	Adjacent habitat	Degraded forest edge	Primary forest interior
*Androlaelaps fahrenholzi*	66 (31.3)	1119 (54)	115 (9)	22 (1.0)
*Ctenophthalmus cabirus*	2 (0.9)	5 (0.2)	8 (0.6)	5 (0.2)
*Dermanyssus gallinae*		1 (0)		3 (0.1)
*Dinopsyllus lypusus*		11 (0.5)	6 (0.5)	33 (1.6)
*Haemaphysalis leachi*	18 (8.5)	98 (4.7)	9 (0.7)	3 (0.1)
*Hemimerus talpoides*			9 (0.7)	66 (3.1)
*Hoplopleura intermedia*	9 (4.3)	90 (4.3)	40 (3.1)	66 (3.1)
*Hoplopluera neumanii*		1 (0)		
*Laelaps nuttalli*	5 (2.4)	538 (26)	1060 (83)	1857 (88.1)
*Leptopsylla segnis*	9 (4.3)	51 (2.4)	1 (1)	1 (0.0)
*Nosopsyllus Incisus*	2 (0.9)	39 (2)	6 (0.5)	4 (0.2)
*Polyplax spinulosa*	31 (14.7)	136 (7)	27 (2.1)	49 (2.3)
*Xenopsylla cheopis*	69 (32.7)		2 (0.2)	
Number of rodent and shrew in each habitat	5	15	14	12
Diversity (Shannon-Weiner's index) of rodents and shrews in each habitat	0.94	1.97	1.67	1.39

In parenthesis is the percentage abundance of each parasite in a given habitat.

**Table 5:** Prevalence of ectoparasites in rodent and shrew species captured along a degradation gradient in Mabira Central Forest Reserve.

Ectoparasite	No. of examined rodents/shrews	No. of rodent/shrew with ectoparasites	Prevalence of rodents (%)	Prevalence of shrew (%)	No. of ectoparasite/individual	Constituent ratio of rodents	Constituent ratio of shrews (*C*)	Index of rodents/shrews	Mean abundance
Flea	1327/84	146/18	11	21.4	225/29	4	33	0.19/0.34	1.75/1.61
Mite	1327/84	809/11	61	13	4746/40	85	45	0.61/0.13	5.87/3.54
Tick	1327/84	57/8	4.3	9.5	117/11	2	13	0.13/0.13	2.05/1.37
Lice	1327/84	98/2	7.4	2.4	441/8	8	9	0.33/0.09	4.5/4.0
Earwig	1327/84	10	0.71	0	77/0	1	0	0.05	7.5
Totals		1159			5692	100	100		4.91

**Table 6:** Percentage prevalence of various ectoparasite types in rodent and shrew species.

	Mite	Flea	Tick	Lice
*Aethomys hindei*	0.46	1.45	0.00	0.08
*Arvicanthis niloticus*	0.38	0.30	0.08	0.08
*Deomys ferrugeneus*	0.08	0.08	0.00	0.00
*Gerbilliscus validus*	0.84	0.38	0.08	0.69
*Hybomys univittatus*	0.69	0.23	0.00	0.15
*Hylomyscus stella*	17.61	0.23	0.00	0.91
*Lemniscomys striatus*	9.07	1.22	1.91	0.99
*Lophuromys ansorgei*	1.52	0.38	0.69	0.23
*Lophuromys stanleyi*	4.50	1.22	0.69	0.84
*Malacomys longipes*	0.08	0.00	0.00	0.00
*Mastomys erythroleucus*	4.34	0.99	0.38	0.69
*Mus minutoides*	0.23	0.00	0.00	0.00
*Mus musculoides*	0.46	0.00	0.08	0.30
*Praomys jacksoni*	16.16	1.37	0.00	1.30
*Praomys misonnei*	0.84	0.53	0.00	0.76
*Rattus rattus*	0.76	2.74	0.46	0.46
*Scutisorex congicus*	2.38	7.14	0.00	0.00
*Crocidura turba*	4.76	4.76	1.19	0.00
*Crocidura luna*	0.00	1.19	0.00	0.00
*Crocidura nigrofusca*	1.19	2.38	0.00	0.00
*Crocidura olivieri*	4.76	5.95	8.33	2.38

**Table 7:** A one-way ANOVA for parasite prevalence and mean infestation.

Variable	Df	*F*/*t* (prevalence/mean infestation)	Prob > *F* (prevalence/mean infestation)
Host sex	1	0.08/10.22	0.779/0.001
Host age		-0.828/0.649	0.022/0.788
Host habitat	3	92.46/14.58	0.001/0.001
Season	1	3.18/10.26	0.075/0.001
Host species	21	68.76/8.28	0.001/0.001

**Table 8:** A log-transformed multiple regression model with dummies for ectoparasite prevalence in rodents and shrews of Mabira Central Forest Reserve.

Source	SS	Df	MS	*F*-ratio	*P* value
Model	1532.5	10	153.25	119.52	0
Residual	1471.95	1149	1.28		
Total	3004.45	1158			
Model results					
Ln (prevalence)	Exp(coef.)	Std. err.	*T*	*P* > *t*	Percentage ±
Host age					
Juvenile^∗^					
Subadult	1.086	0.239	0.38	0.707	8.6
Adult	0.952	0.169	-0.28	0.782	-4.8
Season					
Dry^∗^					
Wet	0.815	0.055	-3.02	0.003	-18.5
Habitat					
Adjacent habitat^∗^					
Primary forest interior	0.435	0.036	-10.06	0.001	-56.5
Degraded forest edge	0.457	0.042	-8.56	0.001	-54.3
Homestead	0.938	0.137	-0.44	0.663	-6.2

^∗^Reference variable per category.

**Table 9:** A log-transformed multiple regression model with dummies for mean infestation in rodents and shrews of Mabira Central Forest Reserve.

ANOVA					
Source	SS	Df	MS	*F*-ratio	*P* value
Model	246.8	9	27.42	57.54	0.001
Residual	547.62	1149	0.48		
Total	792.42	1158			
Model results					
Ln (mean infestation)	Exp(coef.)	Std. err.	*T*	*P* > *t*	Percentage ±
Sex					
Female^∗^					
Male	1.167	0.048	3.79	0.001	16.7
Habitat					
Adjacent habitat^∗^					
Degraded forest edge	0.863	0.048	-2.65	0.008	-13.7
Primary forest interior	0.936	0.047	-1.31	0.19	-6.4
Homestead	1.024	0.091	0.27	0.787	2.4
Season					
Dry^∗^					
Wet	0.88	0.036	-3.1	0.002	-12
Constant	-4.62	0.24	-19.27	0	

^∗^Reference variable per category.

## Data Availability

All data used to support the findings of this study are included within the article in the form of tables; however, if anyone is interested in the raw data (Excel), it is available from the corresponding author upon request.
